# Impact of Thermal Treatment of Nb_2_O_5_ on Its Performance in Glucose Dehydration to 5-Hydroxymethylfurfural in Water

**DOI:** 10.3390/nano10091685

**Published:** 2020-08-27

**Authors:** Katarzyna Morawa Eblagon, Anna Malaika, Karolina Ptaszynska, Manuel Fernando R. Pereira, José Luís Figueiredo

**Affiliations:** 1 Associate Laboratory LSRE-LCM, Faculty of Engineering, University of Porto, Rua Dr. Roberto Frias s/n, 4200-465 Porto, Portugal; karolina.ptaszynska@amu.edu.pl (K.P.); fpereira@fe.up.pt (M.F.R.P.); jlfig@fe.up.pt (J.L.F.); 2 Faculty of Chemistry, Adam Mickiewicz University in Poznań, Uniwersytetu Poznańskiego 8, 61-614 Poznań, Poland

**Keywords:** cascade glucose dehydration, niobium oxide, sugar conversion, Lewis/Brønsted acidity, green chemistry

## Abstract

The cascade dehydration of glucose to 5-hydroxymethylfurfural (HMF) was carried out in water over a series of Nb_2_O_5_ catalysts, which were derived from the thermal treatment of niobic acid at 300 and 550 °C, under air or inert atmosphere. Amorphous niobic acid showed high surface area (366 m^2^/g) and large acidity (2.35 mmol/g). With increasing the temperature of the thermal treatment up to 550 °C, the amorphous Nb_2_O_5_ was gradually transformed into a pseudohexagonal phase, resulting in a decrease in surface area (27–39 m^2^/g) and total acidity (0.05–0.19 mmol/g). The catalysts’ performance in cascade dehydration of glucose realized in pure water was strongly influenced by the total acidity of these materials. A remarkable yield of 37% HMF in one-pot reaction in water was achieved using mesoporous amorphous niobium oxide prepared by thermal treatment of niobic acid at 300 °C in air. The best-performing catalyst displayed a total acidity lower than niobic acid (1.69 mmol/g) which afforded a correct balance between a high glucose conversion and limited further conversion of the target product to numerous polymers and humins. On the other hand, the treatment of niobic acid at 550 °C, independently of the atmosphere used during the sample preparation (i.e., air or N_2_), resulted in Nb_2_O_5_ catalysts with a high ratio of Lewis to Brønsted acid sites and poor total acidity. These materials excelled at catalyzing the isomerization step in the tandem process.

## 1. Introduction

The efficient utilization of lignocellulosic biomass is essential for building a sustainable society. In this respect, cellulose can be converted into glucose via a combination of mechanical treatment and acid-catalyzed hydrolysis [1,2,3]. Subsequently, as-obtained glucose can be further transformed into biofuel precursors [4,5], commodity chemicals [6], or other important platform molecules such as 5-hydroxymethylfurfural (HMF). 

HMF can be obtained by acid-catalyzed dehydration of hexoses such as glucose or fructose. This compound is listed by the U.S. Department of Energy (DOE) as one of the “Top 10 + 4” most important bio-based chemicals in a prospective biorefinery [7]. Moreover, HMF is considered a “sleeping giant” in the field of renewable feedstocks [7], because it can be upgraded with high selectivity to a wide range of industrially important products such as: 2,5-icarboxylic acid (FDCA), which is a monomer used in the production of next generation bio-polyesters [8,9,10], chemicals [3], biofuel precursors, including γ-valerolactone [11], 5-ethoxymethylfurfural (EMF) [12], or 2,5-dimethylfuran [13], among many others. Despite the wide array of applications for HMF, sustainable and economical synthetic routes for its production on an industrial scale are yet to be developed.

The HMF yield obtained via dehydration of carbohydrates depends significantly on the type of sugar molecule used as a substrate. Fructose is generally found to be more reactive and selective towards HMF than glucose [14,15,16]. Thus far, the highest success in the efficient synthesis of HMF has been obtained using fructose as a feedstock and catalysts containing Brønsted acid (BA) sites [7,17,18]. However, fructose is a comparatively expensive substrate, as a result of its costly production (i.e., isomerization of glucose to fructose), being economically limited to only a 42% yield [5]. On the other hand, glucose can be easily obtained from cellulose, thus it is the cheapest and the most abundant sugar, which makes it a desirable raw material for the mass production of HMF [5]. 

The development of an active and selective catalyst for production of HMF from hexoses is rather complex mainly because BA sites catalyze not only dehydration of fructose to HMF, but also side reactions such as rehydration of HMF to levulinic and formic acids [7], and condensation/polymerization of HMF. The latter reactions result in the formation of insoluble materials often called “humins” [19] which tend to deactivate the catalyst [10]. In addition, humins can also be produced by cross-polymerization of glucose and HMF, a reaction catalyzed by Lewis acid (LA) sites [20,21,22]. Most importantly, when the reaction is carried out in water, the negative impact of these side reactions on the yield of HMF is more significant [11,21,23]. Hence, the process is normally carried out in ionic liquids [24,25], high boiling point organic solvents such as dimethyl sulfoxide DMSO [16], or biphasic systems [26,27,28]. However, more restrictive regulations, such as REACH (Registration, Evaluation, Authorization and Restriction of Chemicals) in the European Union (EU), are strong incentive for careful consideration to select the most appropriate alternative solvent in terms of performance, availability and price, taking into account the health and safety as well as environmental impacts [26]. In this sense, neat water seems to be the most attractive solvent for green HMF production. 

The dehydration of glucose to HMF requires a bifunctional catalyst in order to attain acceptable yields of the target product. This is simply because the tandem transformation of glucose to HMF involves two consecutive steps, i.e., glucose isomerization to fructose (catalyzed by a Lewis acid (LA) or a base), followed by dehydration of the in-situ generated fructose to HMF (catalyzed by BA sites). 

Niobic acid shows a high acid strength (H_0_ ≤ −5.6–8.2) [29,30], which is equivalent to >70% H_2_SO_4_ [31], and it is a “water tolerant” catalyst containing two types of active sites [32]. In distorted polyhedrons of Nb_2_O_5_, a part of the surface –OH groups functions as BA sites, while NbO_4_ tetrahedra function as LA sites [33]. As a result, niobium pentoxide (Nb_2_O_5_) and niobic acid (hydrated form of Nb_2_O_5_) serve as highly acidic heterogeneous catalysts that display excellent activity in various reactions [31,32,34,35,36]. In this regard, a variety of niobium-based catalysts has been successfully applied in dehydration of hexoses in biphasic solvent media. For instance, niobia/carbon composites were successfully applied in the conversion of cellulose and glucose to HMF in a THF/H_2_O biphasic system, and over 58% selectivity to HMF was achieved from glucose. Using the same biphasic system and Nb_2_O_5_ catalyst, Li et al. obtained 93% of glucose conversion with 16% yield of HMF at 160 °C [37]. In comparison, much lower yields of HMF were obtained using niobia-based catalysts in dehydration reactions taking place in pure water. Transformation of sucrose and glucose using niobia gave only 17% and 13% yields of HMF, with low sugar conversions [15]. On the other hand, when mesoporous niobium phosphate catalysts were applied, Wang et al. reported 34% yield of HMF with 68% conversion of glucose [38]. Thus far, the best result in dehydration of glucose in water using a niobium containing catalyst was reported in the case of a mixture of niobium acid and niobium phosphate, which achieved 55% of glucose conversion with unprecedented 56% selectivity to HMF [39]. 

Many papers thus far demonstrated that the proper balance of the strength of BA and LA sites is the key feature affecting the yield of HMF obtained using Nb_2_O_5_ materials as catalysts [15,40]. The acid properties of these materials can be controlled by thermal treatments; however, and to our best knowledge, there is no work focusing on the direct impact of calcination/annealing on the catalytic properties of Nb_2_O_5_ catalysts in the cascade transformation of sugars. Thus, in the present work, the changes in the acid properties of Nb_2_O_5_ were studied as a function of temperature and atmosphere of applied heat treatment. The relationship between the concentration of acid sites on these catalysts and their catalytic performance was established in direct dehydration of glucose to HMF carried out in neat water, without addition of organic solvents and in the absence of any additives or promoters.

## 2. Experimental 

### 2.1. Materials Preparation

Niobium acid was prepared according to the procedure presented by M.L. Marin et al. [31] using NbCl_5_ (Merck-Sigma Aldrich, Darmstadt, Germany). Basically, when NbCl_5_ (5 g) was dissolved in de-ionized (DI) water (200 mL), a white precipitate on the bottom of the container, known as niobic acid (hereafter abbreviated Nb_2_O_5_·nH_2_O) was produced, according to the hydrolysis equation shown below. Continuous very strong magnetic stirring was carried on at room temperature for 3 h to complete the reaction.

$$2{\mathrm{NbCl}}_{5}+5H_{2}O={\mathrm{Nb}}_{2}O_{5}+10\mathrm{HCl}$$

Subsequently, the obtained material was washed with DI water using a centrifuge (at 3000 rpm) until the pH of the filtrate was neutral, and it was left to dry overnight in a laboratory oven at 100 °C. The yield of the synthesis was calculated to be around 55%. 

In the following step, the prepared Nb_2_O_5_ ·nH_2_O was thermally treated at 300 °C in air for 2 h (hereafter abbreviated as Nb_2_O_5__300_air), or at 550 °C in air or in N_2_ for 6 h (hereafter abbreviated as Nb_2_O_5__550_air and Nb_2_O_5__550_N_2_, respectively). The gas flow rate used was 150 mL/min and the heating rate applied was 5 °C/min in all of the experiments. 

### 2.2. Materials Characterization 

The specific surface area and pore structure of Nb_2_O_5_ materials were measured by N_2_ adsorption at 77 K in an automatic surface area and porosity analyzer (Quantachrome Nova, Quantachrome Instruments, Boynton Beach, FL, USA). The specific surface area (S_BET_), external surface area (S_meso_), and the total pore volume (V_tot_) were determined applying standard methods.

The total acid sites content in the samples was evaluated by a potentiometric back titration method. Briefly, 0.1 g of a sample was dispersed in 50 mL of a 0.01 M NaOH solution and shaken for 20 h. After filtration, the filtrate was titrated with a 0.05 M solution of HCl under stirring. 

Temperature-programmed desorption of ammonia (NH_3_-TPD) experiments (homemade unit) were performed to further characterize the acidity of the samples. Typically, 100 g of material was placed inside a flow reactor. Subsequently, the sample was heated to 600 °C under He flow with a heating rate of 10 °C/min and kept under these conditions for 0.5 h. In the next step, the system was cooled down to 90 °C and the sample was saturated with NH_3_ for 0.5 h. Finally, the reactor was purged with He, and the amount of desorbed NH_3_ was registered using a TCD detector whilst increasing the temperature of the reactor to 600 °C (with a heating rate of 10 °C/min under He flow).

X-ray diffraction (XRD) patterns of the samples were recorded at room temperature with a step size of 0.05° in the 2 theta range from 10 to 80°, using a D8 Advance Diffractometer (Bruker, Billerica, MA, USA) with a copper Kα1 radiation (λ = 1.5406 Å) and a silicon strip detector LynxEye. 

The combined thermogravimetric and differential scanning calorimetry (DSC) analyses were performed on a DTG-50 thermal analyzer (Shimadzu, Kyoto, Japan). Around 10 mg of each of the materials was heated from 50 °C to 900 °C with a heating rate of 25 °C/min in a flow of dry air. The samples showing significant weight loss were subsequently reanalyzed in more detail using a much slower heating rate of 10 °C/min in order to obtain the DSC results.

The composition, electronic state and the type of Nb and O species in the Nb_2_O_5_-samples were characterized by X-ray photoelectron spectroscopy (XPS). The measurement was completed using a Kratos AXIS Ultra HSA (Kratos Analytical-Shimadzu, Kyoto, Japan) with VISION software for data acquisition and CASA XPS for data analysis. The investigation was carried out with a monochromatic Al K X-ray source (1486.7 eV), operating at 15 kV (90 W), in FAT mode (fixed analyzer transmission), with a pass energy of 40 eV for regions of interest (ROI) and 80 eV for survey. Data acquisition was executed with pressure lower than 1.0 × 10^−8^ mbar using a charge neutralization system. The C1s line at 284.8 eV from adventitious carbon was used for binding energy referencing. The XPS peaks were fitted using a least squares routine with mixed Gaussian-Lorentzian functions in Casa XPS. Shirley baseline was applied as background. In the decomposition of Nb3d peaks, a constraint of 2.80 eV was used for the spin-orbit splitting, and the (Nb3d_5/2_)/(Nb3d_3/2_) peak area ratio was kept constant and equal to the theoretical value of 1.5. 

### 2.3. Catalytic Testing

The dehydration of glucose to HMF was carried out as follows: the catalyst (0.1 g) was placed together with 30 mL of a glucose solution (1.5 wt% in DI water) in a stainless steel reactor (50 cm^3^ in volume) equipped with a pressure gauge, magnetic stirrer and a heating mantle. Subsequently, the reactor was charged with 2.5 bars of N_2_, after being flushed several times. Once the required temperature was reached and the magnetic stirring was started (at 600 rpm), the first reaction sample (at t = 0) was withdrawn, and simultaneously the reaction time was started. After the desired reaction time, the stirring and heating were turned off and the reactor was cooled down. During the reaction, small aliquots of the reaction mixture were withdrawn for analysis using high-performance liquid chromatography (HPLC). The performance of the prepared catalysts was compared to that of the commercial Nb_2_O_5_ purchased from Sigma Aldrich (Merck-Sigma Aldrich, Darmstadt, Germany), hereafter abbreviated as Nb_2_O_5__SA.

Two different HPLC configurations were used for analyses, and thus two separate samples were prepared by dilution with ultra-pure water (UP H_2_O) from each collected mixture (i.e., for analyses of conversion, and selectivity). The amount of glucose and fructose was measured using an Altech OA 1000 organic sugar column and a refractive index detector using the flow rate of 0.5 mL/min of 5 mM H_2_SO_4_. Selectivity to HMF was measured with a Hydrosphere C18 column paired with a UV detector set at 254 nm, using a flow rate of 0.4 mL/min of 40% methanol in UP H_2_O. The calculations of the conversion and selectivity were made based on external calibration curves, accordingly with the equations shown below:

$$\mathrm{Conversion}\;\mathrm{of}\;\mathrm{glucose}\;\left(\%\right)=\;\frac{\mathrm{Amount}\;\mathrm{of}\;\mathrm{glucose}\;\mathrm{converted}\;}{\mathrm{Amount}\;\mathrm{of}\;\mathrm{glucose}\;\mathrm{initially}\;\mathrm{used}\;}\;\times \;100\%$$

$$\mathrm{Selectivity}\;\left(\%\right)=\frac{\mathrm{Amount}\;\mathrm{of}\;\mathrm{the}\;\mathrm{product}}{\mathrm{Amount}\;\mathrm{of}\;\mathrm{glucose}\;\mathrm{converted}\;}\times \;100\;\%$$

## 3. Results and Discussion

### 3.1. Textural Properties

The textural properties of the catalysts are compared in Table 1. As can be seen, the lowest specific surface area (of only 4 m^2^/g) was shown by the commercial Nb_2_O_5__SA, which was mainly associated with the external surface of the crystallites. On the other hand, the synthesized materials exhibited considerably more developed textural features, which were strongly dependent on the applied thermal treatment. In general, all of these materials presented only external porosity (i.e., meso- and macro-pores) without measurable microporosity. The niobic acid (i.e., Nb_2_O_5_·nH_2_O) showed a substantially high specific surface area of 366 m^2^/g. The data in Table 1 confirmed that heat treatments of Nb_2_O_5_·H_2_O resulted in a deterioration of its textural properties, with a substantial decrease in S_BET_ and S_meso_. Total pore volumes of these materials followed the same pattern as that of the surface area. This is probably related to the pore coalescence due to the further crystallization of walls separating mesopores in the material structure [41]. A large reduction in porosity during crystallization of niobium oxide has been previously reported [42,43]. Moreover, the higher the temperature of the treatment, the more significant deterioration of the textural properties was observed. For example, the treatment in air at 300 °C led to 43% reduction in S_BET_, but further increase in the temperature to 550 °C resulted in over 90% drop in S_BET_ as compared to S_BET_ of Nb_2_O_5_·nH_2_O. This is consistent with the increase in particle size due to sintering, measured by XRD (discussed later). Moreover, calcination of the Nb_2_O_5_·nH_2_O at 550 °C caused a larger reduction in S_BET_ (sample Nb_2_O_5__550_air) than annealing at the same temperature in N_2_ (sample Nb_2_O_5__550_N_2_). This may be due to the fact that crystallization of Nb_2_O_5_ (mentioned above) in an inert atmosphere often requires higher temperatures than in an oxidative atmosphere [44], thus Nb_2_O_5__550_air was slightly more crystalline than Nb_2_O_5__550_N_2_. This was confirmed by the XRD results discussed below.

### 3.2. Crystallinity of the Samples

In accordance with the literature findings, Nb_2_O_5_·nH_2_O is in its amorphous state, which can be transformed into low temperature pseudohexagonal niobium oxide (TT-Nb_2_O_5_) upon increasing the temperature up to 300–500 °C [45]. TT-Nb_2_O_5_ is thermodynamically the least stable phase of niobium oxide, and at 600–800 °C it is rapidly transformed into an orthorhombic structure (T-Nb_2_O_5_) [46], followed by monoclinic niobium oxide (H-Nb_2_O_5_) at T > 1000 °C [34,35]. In general, the crystallization conditions of the Nb_2_O_5_ structure depend on the starting material, synthesis method and heat treatment conditions. 

**Figure 1:**
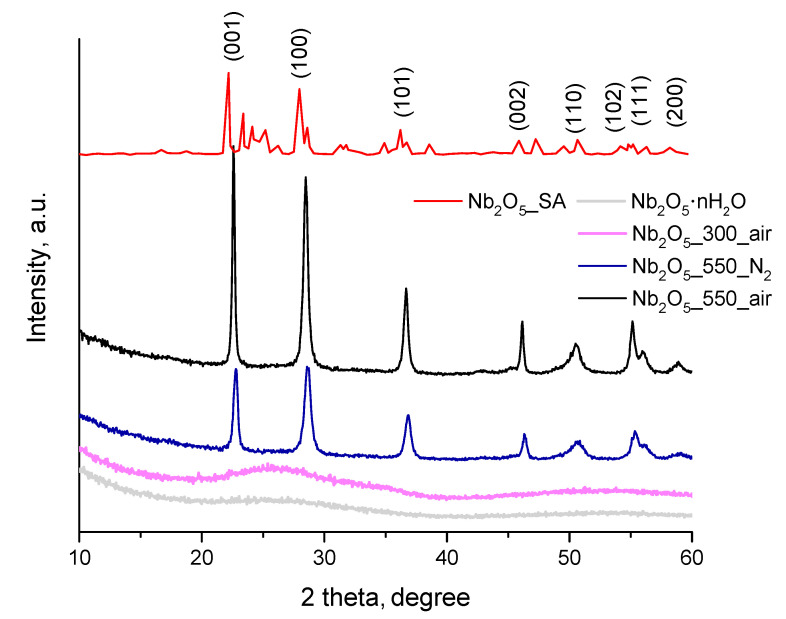
X-ray diffraction (XRD) patterns of as- prepared Nb_2_O_5_·nH_2_O and after different heat treatments in comparison with the X-ray pattern of Nb_2_O_5__SA.

The crystalline structure and crystallinity of the as-prepared materials and that of commercial sample were analyzed by XRD and the results are shown in Figure 1. Considerable differences in the XRD patterns in terms of intensity and shape of the diffraction peaks are clearly visible in this figure. Obviously, the crystallinity of the samples was strongly influenced by the temperature of the treatments. The as-prepared Nb_2_O_5_·nH_2_O without any further post-treatments presented two very broad humps with maxima of around 25° and 53°, which confirmed the amorphous nature of this material. The lack of reflexes in this diffraction pattern can also be related to the presence of structural water [30]. Upon calcination at 300 °C, mild diffraction peaks appeared in the pattern of Nb_2_O_5__300_air, which suggested that the crystallization of niobium oxide was initiated at this temperature. Nevertheless, Nb_2_O_5__300_air still exhibited a mostly amorphous structure, with XRD pattern strongly resembling that of Nb_2_O_5_·nH_2_O (see Figure 1). Further increase in the temperature to 550 °C clearly adjusted the degree of structural order of niobium oxide and peaks typical of TT-niobium oxide phase (JCPDS, 28-317) [35,40,47,48] were observed for both samples (i.e., Nb_2_O_5__550_N_2_ and Nb_2_O_5__550_air), regardless of the atmosphere used during the thermal treatments. On the other hand, as it can be seen in Figure 1, the commercial Nb_2_O_5_ (Nb_2_O_5__SA) has a multiphase character and contains in majority H-niobium oxide phase (ICDD card No 37-1468), together with some T-niobium oxide (ICDD card No 27-1003) and a minor addition of M-niobium oxide (ICDD card No 32-0711) [49,50]. M-Nb_2_O_5_ polymorph has been previously reported to form as a metastable phase during transformation of T-niobium oxide into H-niobium oxide [51].

It should be noted that the increase in crystallinity with the temperature of the thermal treatment, confirmed by the diffraction patterns, was accompanied by a decrease in S_BET_ of the thermally treated samples (compare results in Table 1 and Figure 1). Moreover, it can be noticed in the aforementioned figure that the annealed sample showed slightly broader peaks than its calcined counterpart, which suggests more pronounced crystallinity and/or larger crystallite size of the latter [52]. This observation, together with the higher S_BET_ of Nb_2_O_5__550_N_2_ compared to that of Nb_2_O_5__550_air (see Table 1), indicates that indeed higher temperatures may be needed for crystallization of niobium oxide under inert atmosphere. 

Average crystallite sizes of the crystalline Nb_2_O_5_ samples were calculated applying the Scherrer equation, and slightly larger crystallites were estimated in the case of calcined Nb_2_O_5_ as compared to its annealed counterpart (i.e., 24 nm for Nb_2_O_5__550_air and 17 nm for Nb_2_O_5__550_N_2_). These results are in line with the surface area measurements of these samples (see Table 1). An average crystallite size of 58.5 nm was obtained in the case of Nb_2_O_5__SA, which agrees with other reports [53], and goes in line with a much lower S_BET_ of this sample as compared to the other results (see Table 1). 

Overall, it can be concluded that the temperature of the thermal treatment exerted stronger influence on the ordering of the Nb_2_O_5_ structure than the type of gaseous atmosphere (i.e., N_2_ or air).

### 3.3. Chemical Properties

#### 3.3.1. X-ray Photoelectron Spectroscopy (XPS) Studies

The XPS survey scans of all of the prepared materials showed the presence of Nb, O and C, similarly to that of Nb_2_O_5__SA. The amount of adventitious carbon found on the surface of these samples was between 14.7 and 18.7 at%. The chemical composition of the catalysts, excluding carbon, is gathered in Table 2. The atomic ratios of O/Nb (oxide stoichiometry) were measured for all samples on the basis of the area under the peak component of O1s and that of Nb3d (Nb3d_5/2_). As can be seen from these results, all samples showed O/Nb atomic ratios higher than the nominal value of 2.5, suggesting that these samples were oxygen-rich on the surface. Furthermore, comparing the values of O/Nb, it is clear that the amount of oxygen decreased with the increase in the temperature of the thermal treatment of Nb_2_O_5_·nH_2_O. Thus, values closer to the nominal ratio were obtained by samples treated at 550 °C and in the case of Nb_2_O_5__SA (calcined at high temperatures). The changes in the ratio can be attributed to gradual dehydration of the Nb_2_O_5_·nH_2_O material during its thermal treatment, leading to a decrease in the amount of hydroxide on the surface of the resulting samples. This observation is further supported by analysis of the high resolution O1s spectra of these materials, discussed below.

**Figure 2:**
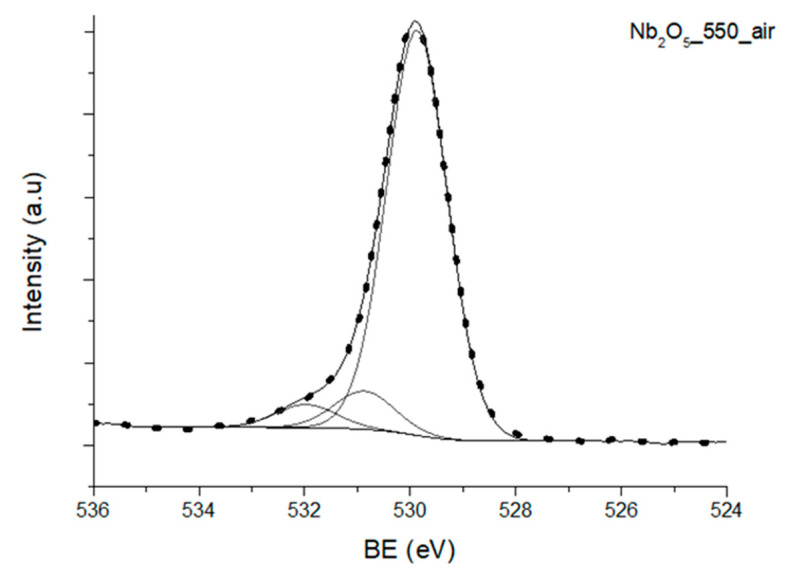
High-resolution XPS scan of the O1s region of Nb_2_O_5__550_air.

The representative fit of the O1s region of Nb_2_O_5__550_air is shown in Figure 2. As can be seen in this result, the high resolution O1s XPS region was deconvoluted into three different species, namely O (I) with BE of 529.80–530.59 eV, assigned to lattice oxygen (O^2-^) in metal oxide, O (II) with BE of 530.87–531.63 eV [42,52], corresponding to oxygen in CO and OH [52,54], and O (III) with BE of 531.97–532.83 eV [47,54] attributed to physically adsorbed water. It is noted that the C-O interactions probably originated from the presence of adventitious carbon in all samples, as previously mentioned. 

The contribution of different oxygen species to the O1s region of the studied samples, and the ratio between adsorbed oxygen species on the surface of the materials (including hydroxide and the lattice oxygen), O(II)/O(I), can be found in Table 3. It is clear from the presented data that O(II)/O(I) is lower for the crystalline samples, which were treated at higher temperatures. Thus, these data support the fact that the samples were dehydrated at higher temperatures, which resulted in the lower amount of oxygen on their surface.

**Figure 3:**
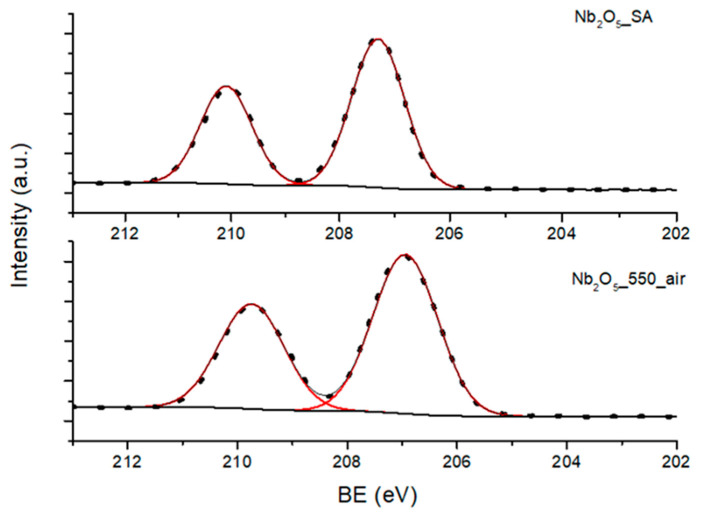
High-resolution Nb3d regions of Nb_2_O_5__SA and Nb_2_O_5__550_air.

Comparison of the XPS Nb3d regions of Nb_2_O_5__SA and Nb_2_O_5__550_air can be found in Figure 3. The high-resolution core level XPS spectra of Nb3d, due to spin-orbit splitting, showed two photoelectron peaks (Nb3d_5/2_ and Nb3d_3/2_) with BE in the range of 206–207 eV and 209–210 eV, corresponding to Nb_2_O_5_ [54]. Moreover, a higher BE of the core level of the Nb3d main peak (Nb3d_5/2_) was recorded for the amorphous samples, and the BE values decreased with increasing crystallinity of the materials, as evidenced in Figure 3. For example, a BE of 207.62 eV was measured for Nb_2_O_5_·nH_2_O, as compared to a BE of 206.94 eV recorded for Nb_2_O_5__550_air. Thus, there is a clear shift in the oxidation state of Nb from partially Nb^4+^ to mostly Nb^5+^ in Nb_2_O_5_, which was previously reported by Tsang et al. [55]. This shift in BE is directly connected with a strong lattice deformation in niobium oxide taking place during thermal treatment [56], which is due to the progressive change in the chemical environment of the niobium atoms.

#### 3.3.2. Acidity of the Catalysts

As described in the introduction, the dehydration of fructose (i.e., the second step in glucose dehydration to HMF) is an acid-catalyzed process, which depends on the amount and type of acid sites available on the surface of the catalyst. The total amount of acid sites (A_tot_) was estimated for all samples, and the results are gathered in Table 2. The synthesized materials are clearly more acidic than their commercial counterpart (Nb_2_O_5__SA). Moreover, the acidity was dependent on the thermal treatment applied. Trace levels of acidic sites (A_tot_ = 0.02 mmol/g) were found on a commercial Nb_2_O_5__SA which was probably treated at a high temperature, judging by its crystalline phase. In contrast, the total content of acid sites measured for the untreated sample (i.e., Nb_2_O_5_·nH_2_O) was much higher and equal to 2.35 mmol/g. In this case, strong Brønsted acidity (BA) is expected, due to the proton generation from the water molecule on the exposed Nb^5+^ [46]. Moreover, the BA sites should decrease with an increase in temperature of the thermal treatment [47], which was indeed observed. This is the result of the continuous dehydration of Nb_2_O_5_·nH_2_O during the thermal treatments applied, which was evidenced earlier by our XPS results (see Table 2 and Table 3). Remarkably, the acidity decreased more significantly when the thermal treatment was carried out under inert atmosphere than in the presence of air (compare samples Nb_2_O_5__550 air and Nb_2_O_5__550_N_2_ in Table 2). It was previously reported that niobic acid (Nb_2_O_5_·nH_2_O) calcined at moderate temperatures of 100–300 °C shows an acidic character, although it becomes almost neutral when calcined at 600 °C, which is in line with our results [47]. On the other hand, various surface LA sites are expected to be created at elevated temperatures due to the exposed cations in close proximity to oxygen vacant sites on the surface of Nb_2_O_5_ [46,47].

**Figure 4:**
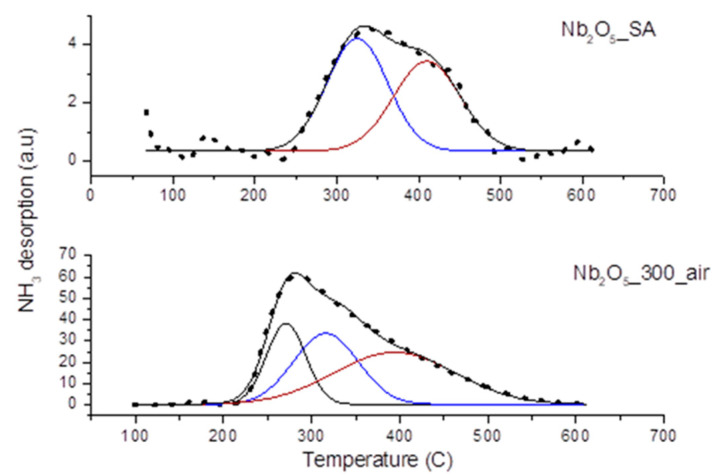
NH_3_-TPD (temperature-programmed desorption) curves of Nb_2_O_5__SA and Nb_2_O_5__300_air together with the fitting of the quantity of weak, medium and strong acid sites. Note the large difference in values on y-axis between both curves.

The strength and number of acid sites of the selected samples, namely Nb_2_O_5__SA and Nb_2_O_5__300_air, were additionally characterized by the NH_3_-TPD technique, and the results are shown in Figure 4. As can be observed, the NH_3_ desorption peaks for both samples were measured in similar temperature regions (i.e., roughly 210 to 550 °C). The broad signal in this region suggests the co-existence of multiple poorly resolved peaks. It is noted that the signals for both samples significantly differed in their intensity (note the values on the y-axis in Figure 4), as well as in the position of the peak maxima and the overall shape of the desorption curve. The intensity of the curves suggests that Nb_2_O_5__SA displayed considerably lower acidity than Nb_2_O_5__300_air, which agrees well with the total acidity measurements shown in Table 2. In fact, the total amount of desorbed ammonia from the commercial sample was only 18 µmol/g, as compared to 216 µmol/g found for Nb_2_O_5__300_air. A multiple-Gaussian function was selected for fitting the experimentally obtained curves in order to find the amount and strength of the acidic sites present in these samples (see Figure 4). 

According to the published literature, the acidic strength of active sites on the materials can be classified depending on the desorption temperature of NH_3_ upon heating the sample under inert atmosphere [40,57]. Thus, the desorption of NH_3_ in the range of 150–250 °C describes weak acid sites, 250–350 °C belongs to medium acid sites and above 350 °C corresponds to strong acid sites [57,58]. The results depicted in Figure 4 demonstrate that Nb_2_O_5__SA contained medium and strong acid sites, whereas weak, medium and strong acid sites were present on Nb_2_O_5__300_air. The acid site distribution as well as the temperature of the peak maxima (T_max_) are listed in Table 4. From the presented data, it can be seen that the commercial sample had medium (53%) and strong sites (47%), whereas Nb_2_O_5__300_air showed strong sites (45%), followed by medium and weak sites (33 and 22%), respectively. These results suggest that the weak acid sites on Nb_2_O_5_ are reduced more severely at higher calcination temperatures than their stronger counterparts.

Even though it is not possible to unambiguously distinguish or quantify the amount of LA and BA sites present on these catalysts based on the NH_3_-TPD results, some authors assign the peaks appearing at the low temperature in the TPD profile (below 300 °C) to ammonia desorbing from protonated NH^4+^ cations, which can suggest the presence of BA sites. On the other hand, the peaks appearing at higher temperatures (above 300 °C) are often designated as LA sites [59,60,61]. Thus, our results show substantial differences between the characterized materials, namely that the commercial Nb_2_O_5_ (Nb_2_O_5__SA) displayed LA sites in larger proportion, whereas Nb_2_O_5__300_air exhibited both BA and LA sites. The absence of BA in the commercial sample agreed well with the literature reports [38].

### 3.4. Thermogravimetric Analysis

**Figure 5:**
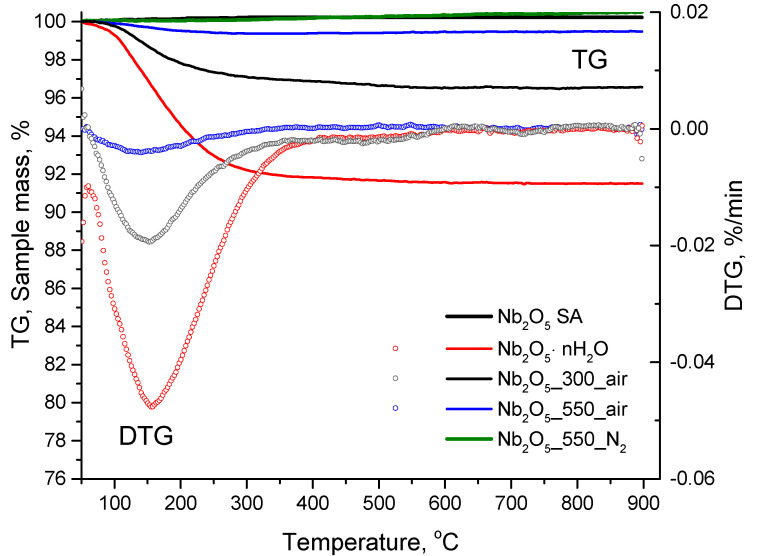
Thermogravimetric analysis of the samples together with the respective derived thermogravimetry (DTG) curves.

The weight loss profiles obtained for the series of Nb_2_O_5_ under nitrogen atmosphere (thermogravimetric analysis, TGA), as well as their respective derivatives (derived thermogravimetry, DTG), are depicted in Figure 5.

As can be seen from these results, Nb_2_O_5_·nH_2_O suffered a 10% weight loss between 50–350 °C, which can be attributed to the elimination of adsorbed and structural H_2_O (i.e., dehydration of the structure) [47,62]. In a similar temperature range (50–280 °C), nearly 5% weight loss was observed for Nb_2_O_5__300_air as compared to only 0.8% in the case of Nb_2_O_5__550_air, with the maximum weight losses at 127 °C and 122 °C, respectively. No further weight loss was observed at higher temperatures for these samples. In contrast, commercial Nb_2_O_5__SA and Nb_2_O_5__550_N_2_ did not show any detectable weight loss up to 900 °C. 

The DTG curves were recorded for the less thermally stable samples (i.e., Nb_2_O_5_·nH_2_O, Nb_2_O_5__300_air and Nb_2_O_5__550_air), and the results are depicted in Figure 5. The profiles show that in all cases the fastest weight loss occurred at about 150 °C. This could be attributed to the partial dehydration of these materials as mentioned before [34,63].

**Figure 6:**
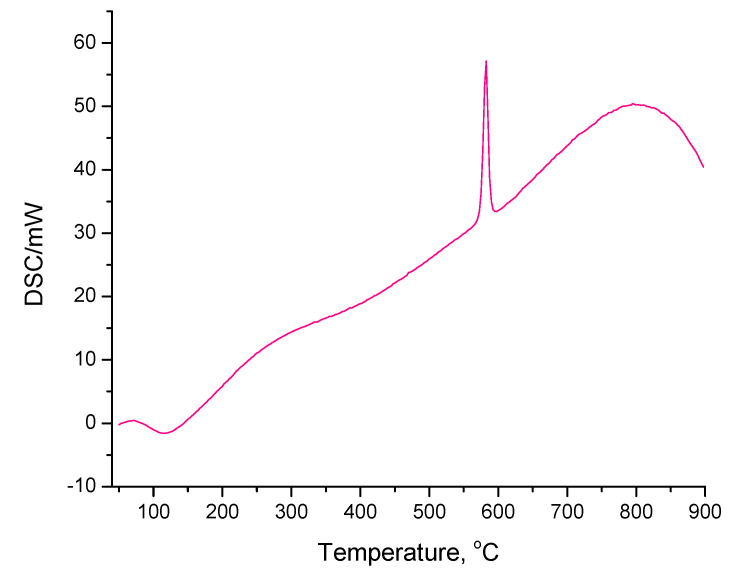
Differential scanning calorimetry (DSC) curve recorded for Nb_2_O_5_·nH_2_O showing the exothermic peak at the temperature of the Nb_2_O_5_ crystallization.

The combined thermogravimetric differential scanning calorimetry (TGA-DSC) of Nb_2_O_5_·nH_2_O and Nb_2_O_5__300_air displayed exothermic peaks at 582 and 587 °C, respectively, which can be attributed to the crystallization of the quasi amorphous samples to the TT crystalline phase [42,64]. The representative DSC curve recorded for Nb_2_O_5_·nH_2_O is shown in Figure 6. A similar result was obtained for Nb_2_O_5__300_air. The exothermic peak observed here was not found in the TGA-DSC analyses of the remaining samples, which supports our XRD results showing that these samples had crystalline nature.

### 3.5. Catalytic Results

#### Effects of the Reaction Time and Temperature on the Dehydration of Glucose to 5-Hydroxymethylfurfural (HMF)

**Figure 7:**
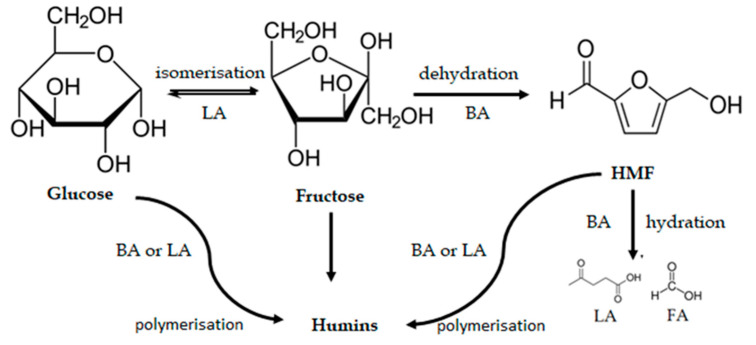
Reaction scheme of one-pot dehydration of glucose to 5-hydroxymethylfurfural (HMF) together with the role of Lewis and Brønsted acid (abbr. LA and BA, respectively) sites.

Niobium oxide catalysts were further evaluated in the conversion of glucose to HMF in pure water in order to identify the influence of the acid sites on the cascade dehydration of glucose to HMF. The reaction scheme is shown in Figure 7. As can be inferred from this figure, the process very often involves side reactions such as rehydration of HMF to levulinic acid (LA) and formic acid (FA) as well as production of soluble and insoluble polymers.

The dehydration of carbohydrates is significantly influenced by the temperature, which affects the yield and selectivity to HMF [65,66]. A thorough review of the literature on the subject displayed a broad range of conditions employed in the dehydration reactions, with the most frequently applied temperatures in the range of 120 to 200 °C [26] and different reaction times between 15 min and 24 h [65]. Thus, prior to testing the prepared catalysts, the influence of the temperature and reaction time was studied using Nb_2_O_5__SA.

**Figure 8:**
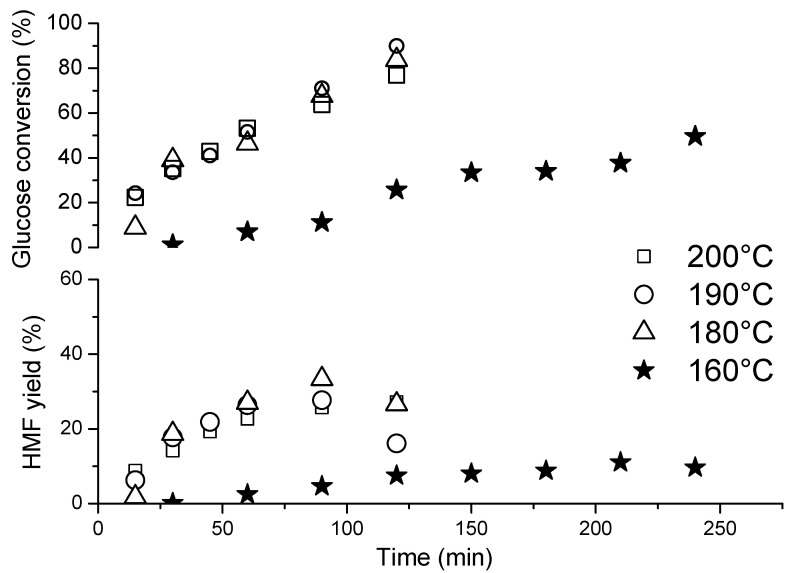
The changes in glucose conversion (top) and the yield of HMF (bottom) versus time over Nb_2_O_5__SA. Reaction conditions: 30 mL of 1.5 wt% solution of glucose in ultra-pure (UP) H_2_O, *p* = 2.5 bar of N_2_, 0.1 g of a catalyst.

The influence of the reaction temperature on the conversion of glucose and on the yield of HMF is shown in Figure 8. Only a very low conversion of glucose (11%) was obtained at 160 °C in 2 h. Thus, the time of the reaction was extended to 4 h; nevertheless, the increase in glucose conversion was not substantial and reached only 38% despite the prolonged reaction time. Moreover, the highest yield of HMF obtained at this temperature was only 11% in 4 h; however, it seems to be rather stable with increasing reaction time (past 120 min) and with further conversion of glucose. Clearly, the temperature of 160 °C was not sufficiently high to obtain acceptable yields of the target product in less than 4 h and under our reaction conditions (i.e., reactor type, pressure, stirring speed, etc.). As a result, the reaction temperature was increased to 180 °C, which caused a substantial increase in glucose conversion to 84% achieved in only 2 h. This was also accompanied by a considerable increase in the maximum yield of HMF from 11% recorded at 160 °C (in 4 h) to 33% (in just 90 min) recorded at 180 °C. Further rise in temperature to 190 °C led to an additional small increment in glucose conversion to about 90%; however, the maximum yield of HMF decreased to 27% at this temperature. In general, the kinetic profiles shown in Figure 8 evidence that the glucose conversion increases with both time and temperature; however, the changes in the maximum yield of HMF followed a volcano curve which already started to decrease at 190 °C. Furthermore, when the temperature was set at 200 °C, the Nb_2_O_5__SA catalyst lost some activity, and the conversion of glucose attained after 2 h was only 77%, as compared to 90% measured at 190 °C. A careful analysis of the data in Figure 8 shows that the conversion obtained in the first hour of the reaction at 200 °C was similar to that obtained at 190 °C. However, in the extended reaction time, a significant decline in the conversion of glucose took place, as compared to the expected value based on the profiles recorded at lower temperatures. Moreover, humins and other polymeric matter were visible on the bottom of the reactor after the reaction was completed at T > 180 °C. In fact, at 200 °C, the production of humins was so severe that it provoked blocking of the filter in the reactor. On the other hand, there was virtually no change in the yield of HMF with an increase in the temperature from 190 to 200 °C, as evidenced in Figure 8. Overall, a careful analysis of the changes in HMF yield with the temperature shown in the aforementioned figure revealed that the yield reached a “peak value” (in 90 to 120 min) when the temperature was higher than 160 °C, which was followed by its immediate decrease with the increase in conversion of glucose. Therefore, the reaction time of 2 h was the optimal time to afford the maximum yield of HMF. Beyond this time, the stability of HMF decreased at T > 160 °C. Since the decline in yield towards HMF did not correspond to the increase in concentration of small organic acids (see Figure 7, rehydration of HMF to FA and LA), it seems that HMF must have been converted to other products such as humins. Thus, the observed deactivation of the catalyst at 200 °C was probably associated with the deposition of carbonaceous matter on the catalyst surface, blocking its active sites. Catalyst deactivation in glucose dehydration caused by carbon deposition was previously reported in the case of zirconium doped silica [67] and Nb-zeolites [59].

**Figure 9:**
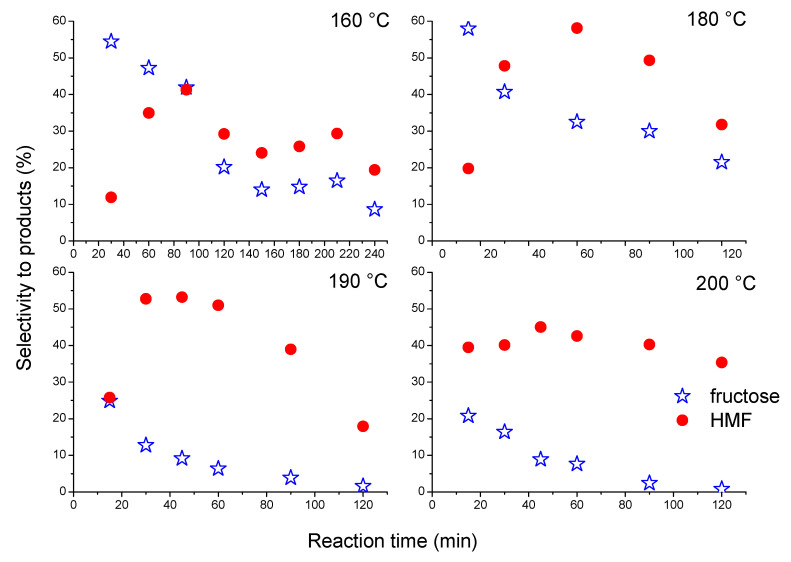
Changes in selectivity to fructose and to HMF with time recorded at different temperatures. Reaction conditions: 30 mL of 1.5 wt% solution of glucose in UP H_2_O, *p* = 2.5 bar of N_2_, 0.1 g of a catalyst.

The changes in selectivity towards fructose and HMF at different reaction temperatures over Nb_2_O_5__SA are shown in Figure 9. It may be observed that at lower temperatures (i.e., 160 °C and 180 °C) fructose was the dominant product at the beginning of the reaction (selectivity of about 60%). This can suggest that glucose is rapidly isomerized into fructose over LA sites on the Nb_2_O_5__SA catalyst (maximum fructose selectivity was obtained in t = 30 min of the reaction). Under more severe conditions (temperatures of 190 °C and 200 °C), the initial selectivity towards fructose was significantly lower, which can mean that at these temperatures in the first 20 min fructose was transformed quickly to other products (HMF and/or humins). Furthermore, the decrease in fructose concentration with time resulted initially in an increase in the concentration of HMF in the whole temperature range studied (i.e., up to about 60–80 min). This indicates that fructose was a primary product of glucose dehydration and HMF was a secondary product of the reaction. This observation supports the fact that the reaction of HMF formation from glucose proceeds via aldose-ketose isomerization between glucose and fructose, involving a hydrogen transfer step and subsequent dehydration of fructose to HMF [15,68], as opposed to a mechanism of stepwise dehydration of glucose via 3-deoxyglucosone proposed by some authors [69]. Furthermore, significant amounts of fructose (20–60%) formed in the initial period of the reaction (i.e., first measured point) also support the argument that glucose conversion to HMF using the Nb_2_O_5__SA catalyst requires the formation of fructose as an intermediate product. The large initial concentration of fructose obtained over the Nb_2_O_5__SA is the result of the presence of mostly LA sites on its surface (as shown by our TPD results, see Table 2), which catalyze the isomerization of glucose to fructose [15,33] (see Figure 7). The accumulation of fructose observed in the reaction mixture is due to a far lesser amount of BA sites present on this catalyst, which facilitate the dehydration step of the cascade process [70]. On the other hand, the increasing amount of HMF at 190–200 °C is mainly the result of the high temperature applied (i.e., hydrothermal conditions), as fructose dehydration to HMF is an autocatalytic process [65].

Furthermore, as can be noticed in Figure 9, the formation of fructose is more favored at lower temperatures (160–180 °C), which can suggest that glucose is rapidly isomerized into fructose over LA sites on the Nb_2_O_5__SA catalyst (maximum fructose selectivity was obtained in t = 30 min of the reaction); however, its further dehydration requires higher temperatures. Moreover, the selectivity to both fructose and HMF (compared at similar conversion of glucose) decreased with increasing reaction temperature from 180–200 °C (compare Figure 8 and Figure 9, at t = 90 min). Thus, the relative rates of isomerization and dehydration reactions change with temperature, which suggests that the most important reactions (i.e., isomerization of glucose, dehydration of fructose and production of humins) probably have different activation energies over the Nb_2_O_5__SA catalyst. The decrease in selectivity to the main products (i.e., fructose and HMF) with increasing reaction temperature also indicates that the formation of by-products in the cascade dehydration of glucose to HMF can be more pronounced at higher temperatures. 

Generally, the stability of HMF decreased with the increasing reaction temperature and time. This was manifested by the drop in selectivity towards the target product, as depicted in Figure 9. The most severe degradation of HMF to side products occurred at 190 °C, where the selectivity to HMF began to drop past the first 30 min of the reaction. However, the reaction time had a negative impact on the selectivity to HMF even at the lowest temperature studied here (i.e., 160 °C), where the concentration of HMF was slowly diminishing past 90 min of the reaction (see Figure 9). Thus, the drop in selectivity to HMF with increasing time at 200 °C was expected to be even steeper than that recorded at lower temperatures. However, only a slow decrease in the selectivity to the target product was observed with the progress of the reaction at the highest temperature (see Figure 9). This result is in line with the fact that some of the active sites on Nb_2_O_5__SA, including those active in degradation of HMF, were blocked at this temperature. However, further transformation of fructose to side-products was observed at 200 °C with time.

Concerning the side products of the reaction, only some small amounts of levulinic and formic acids were found in HPLC analyses of the reactions mixtures (yields below 4%), which suggests that Nb_2_O_5__SA was unable to effectively rehydrate HMF, contrary to the previous reports dealing with heterogeneous catalysts applied in aqueous environment [14,71]. This is related to the lack of substantially strong BA sites on the surface of Nb_2_O_5__SA. In fact, over 80% of the HMF degradation products were accounted by formic acid and levulinic acid in the case of catalysts containing mainly BA sites [22]. However, in our case, as shown by the TPD results, Nb_2_O_5__SA displayed a very low total acidity of only 18 µmol/g (see Table 2), with a clear predominance of LA sites. Thus, the main side products detected in the present work were polymeric black solids (insoluble humins), the formation of which was facilitated at higher temperatures and by higher conversions of glucose, which could indicate that there is a competition between glucose and HMF for these sites on the catalyst. Since glucose contains reactive reducing groups, there is also an increased possibility of cross-polymerization of the substrate with HMF and other reaction intermediates. As mentioned in the introduction, regardless of the type of a catalyst used, the presence of water promotes undesirable fragmentation and condensation reactions between HMF and the carbohydrates present in the reaction mixture. For instance, condensation of glucose to humins was linked to isolated LA sites on the catalysts [70,72].

Overall, the influence of the reaction temperature proved to be critical for the process, giving very low HMF yields at 160 °C and making the catalytic system more efficient at higher temperatures. The best yield of HMF (33%) with substantial conversion of glucose (68%) was obtained at 180 °C (as depicted in Figure 8), and only moderate amounts of humins were produced at this temperature, as compared to the reactions at 190–200 °C (selectivity to by-products of about 21% vs. 55%). Moreover, 2 h was chosen to be the optimum time to achieve the highest yield of HMF before its further degradation to side products. Therefore, the screening of the series of prepared niobium oxide catalysts was carried out at 180 °C for 2 h. 

As previously mentioned, there is a co-existence of different acidic sites on the prepared catalysts, which can significantly affect the rate of HMF production and its further conversion. Thus, it is difficult to get a full picture of the catalytic activity of the selected materials by comparing their performance only at one stipulated reaction time and under the same reaction conditions. Therefore, the catalytic results in the present work were compared at two characteristic points: I) in the first 30 min (i.e., at the beginning of the reaction, preferred point for “more active” materials) and after 90 min of the reaction (the maximum time after which the selectivity to HMF started to decrease for all materials studied). 

A blank experiment (i.e., without a catalyst) showed only 24% conversion of glucose with 53% selectivity to HMF (i.e., 12% yield of HMF) in 90 min of the reaction. This result can be attributed to the enhancing effect of H_3_O^+^ acting as BA, which was undoubtedly present in a low concentration under the selected reaction conditions. Moreover, significant amount of humins were produced in the absence of a catalyst, which shows that the hydrothermal conditions favored polymerization/condensation of HMF and hexoses (see Figure 7).

**Figure 10:**
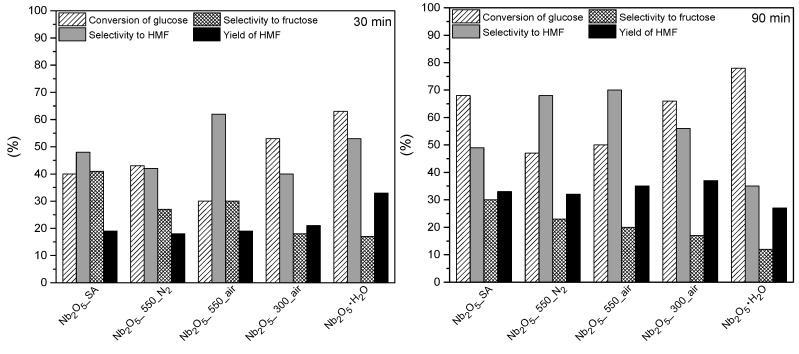
Comparison of the catalytic performance of the niobium oxide catalysts obtained in 30 min (on the left) and in 90 min (on the right) in cascade dehydration of glucose to HMF. Reaction conditions: 30 mL of 1.5 wt% solution of glucose in UP H2O, *p* = 2.5 bar of N2, 0.1 g of a catalyst, T = 180 °C, stirring speed = 400 rpm.

In general, all of the tested materials were active in glucose dehydration to HMF. The catalytic results are depicted in Figure 10. The conversion of glucose together with the yield towards HMF increased in the presence of the catalysts, as compared to the blank experiment. This confirms that the BA sites (H^+^) on niobium oxides preserve their activity in water and probably no significant amount of BA adducts with water (H_3_O^+^) were formed, even under the hydrothermal conditions applied. 

The results obtained in the initial reaction time of 30 min show that the yield of HMF increased in the order: Nb_2_O_5__550_N_2_ (18%) < Nb_2_O_5__550_air (19%) < Nb_2_O_5__SA (19%) < Nb_2_O_5__300_air (21%) < Nb_2_O_5_·nH_2_O (33%). Analyzing the changes in conversion of glucose, it can be concluded that, generally, the conversion decreased with increasing temperature of the thermal treatment of the catalysts (i.e., with their decreasing acidity). This is in line with the previous reports suggesting that the conversion of glucose in water is favored by more acidic catalysts [19]. 

**Figure 11:**
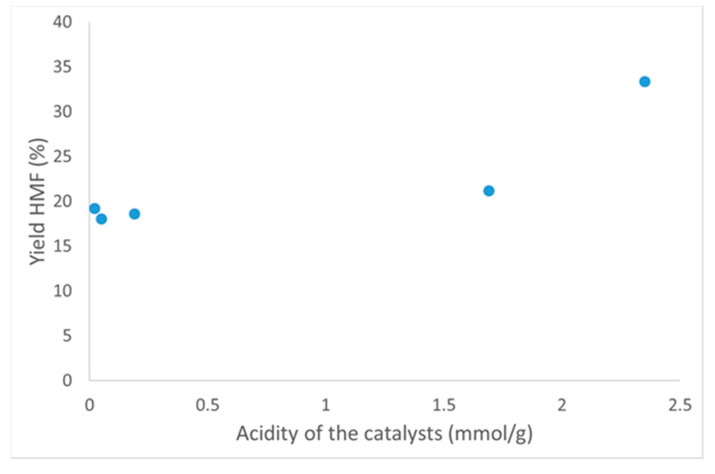
Correlation of the total acidity of the catalysts with the obtained yield of HMF in the initial 30 min of the reaction.

Analyzing the results obtained in the first 30 min of the reaction, no direct relationship between obtained selectivity to HMF and the total acidity of the materials (measured by acid-base titration technique) could be found. In fact, the highest values were obtained by catalysts with quite different acidities (compare Nb_2_O_5__550_air and Nb_2_O_5_·nH_2_O in Table 2 and Figure 10). Interestingly, the selectivity to HMF decreased for some catalysts as compared to the value obtained in the blank experiment. This could result from the presence of some active sites on the surface of the catalysts, which caused the degradation of HMF. On the other hand, the yield of HMF obtained could be correlated with the total acidity of the catalysts, and the results are depicted in Figure 11.

The yield of HMF was also correlated with the S_BET_ of the catalysts, and higher amounts of HMF were produced by the catalysts with larger S_BET_ (see Figure S1).

Moreover, the selectivity to fructose was related to the total acidity of the catalysts and to their S_BET_ (see Figure S2 in Supporting materials). Last but not least, the atomic ratio of Nb to O from XPS (listed in Table 2) was found to have influence on the selectivity to fructose, and the higher the value of Nb/O of the catalyst, the lower the selectivity to fructose was observed in the reaction (see Figure S3 in Supporting materials). The aforementioned correlations were found valid for the data obtained in 30 min and in 90 min of the reaction.

The amorphous niobic acid catalyst with the strong LA together with BA sites [15,47,49] and the highest total acidity among all the materials studied here (see Table 2) presented a higher glucose conversion than the well crystallized Nb_2_O_5_ with TT phase in the first 30 min of the reaction. Clearly, the amorphous nature of Nb_2_O_5_·nH_2_O is structurally different from the crystalline one (i.e., samples treated at higher temperatures e.g., Nb_2_O_5__550_air). It was previously reported that amorphous Nb_2_O_5_ appears to possess disordered corner shared octahedra with only terminal hydroxyls, which are weaker BA sites [19]. These sites can effectively catalyze fructose dehydration to HMF, thus removing fructose from the reaction mixture (see results for niobic acid after 30 min in Figure 10). This, in turn, shifts the reaction path towards the conversion of glucose to fructose (i.e., production of fructose). In fact, there is a clear correlation between acidity of catalysts and selectivity to fructose, namely “more acidic” catalysts showed lower selectivity to fructose, which is in line with the fact that these samples contained more BA sites which were active in fructose dehydration to HMF (second step of the cascade process).

With the extended reaction time to 90 min, the order of the catalysts changed as compared to that obtained in the initial 30 min, and the best result was achieved by Nb_2_O_5__300_air (HMF yield of 37%). Actually, this was the highest yield of HMF attained in the present work. The best catalysts (i.e., Nb_2_O_5__300_air) displayed a lower acidity than that of Nb_2_O_5_·nH_2_O, but apparently this was sufficient to obtain a much higher selectivity to HMF (56%) than niobic acid (35%) in 90 min reaction, while simultaneously limiting further degradation of HMF to humins. Even though the total acidity of the best performing catalyst was significantly lowered by the thermal treatment (from 2.36 mmol/g of Nb_2_O_5_·nH_2_O to 1.69 mmol/g, as shown in Table 2), Nb_2_O_5__300_air most likely contained an optimized ratio of BA to LA sites. It is known that the thermal treatment decreases BA to a greater extent than LA sites [33]. Thus, even though the BA sites on niobium oxide are typically known to give low yields of HMF in glucose dehydration (by promoting HMF transformations) [23], our results showed that the removal of some of these sites from niobic acid by thermal treatment leads to a catalyst rendering moderate yields of the target product, even in pure water. Moreover, although the results obtained in the present work may not seem outstanding (37% yield of HMF, with 66% conversion of glucose in 2 h at 180 °C), they look promising when compared with other works, especially those carried out in water, in the absence of any promoters and using Nb containing catalysts (as described in the introduction). 

**Figure 12:**
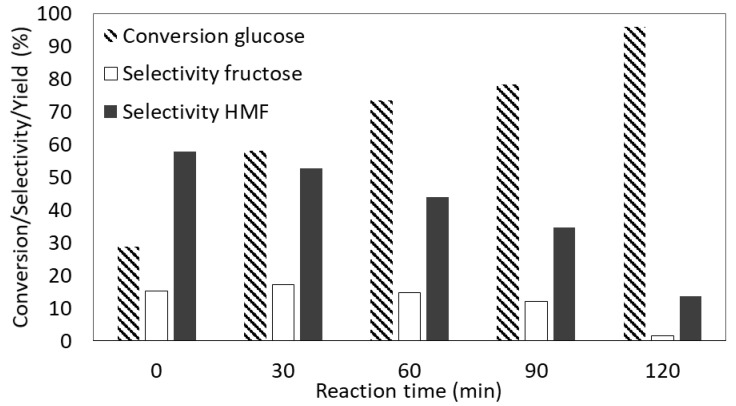
The catalytic performance of Nb_2_O_5_·nH_2_O. Reaction conditions: 30 mL of 1.5 wt% solution of glucose in UP H_2_O, *p* = 2.5 bar of N_2_, 0.1 g of a catalyst, T = 180 °C. Note: the difference in selectivity to 100% in liquid soluble products is given by amount of low molecular mass carboxylic acids, unrecognized products (via high-performance liquid chromatography, HPLC) and solid polymers (humins).

The catalytic performance of the material with the highest total acidity (Nb_2_O_5_·nH_2_O) versus time is presented in Figure 12. The similar bell-shaped profile of HMF yield was shown by all the tested catalysts. Notably, the HMF yield first increased with time together with glucose conversion to a certain maximum value (i.e., maximum yield of HMF), which was followed by a steady decrease of selectivity to HMF with a further increase in glucose conversion. In most cases, the decrease in HMF yield started between 60 and 120 min of the reaction, which was when HMF decomposition surpassed its formation. This could be the result of the competition for the same active sites between glucose and HMF from the beginning of the reaction, as already mentioned. Thus, HMF accumulates in the reaction mixture as long as there is a large concentration of the substrate remaining in the solution. When most of the glucose is converted, more active sites become available and the concentration of HMF starts to decrease. Even though both types of acid sites can be responsible for the production of humins, it seems from our results that a faster drop in HMF yield occurs on the more acidic catalysts. This suggests that BA rather than LA sites were responsible for HMF degradation. As can be seen in Figure 12, when the reaction was carried out using Nb_2_O_5_·nH_2_O, due to the large surface area for proton transfer (see Table 1) and the high density of acid sites available on this catalyst (see Table 2), the selectivity to HMF started to decrease right after the first sample was taken (i.e., right after T = 180 °C was reached). Interestingly, comparing the selectivity to HMF at the beginning of the reaction (i.e., in the first 30 min) and at the end of the reaction (after 90 min), as shown in Figure 10, it is clear that the selectivity to HMF increased monotonically with the reaction time for almost all tested materials. However, in the case of Nb_2_O_5_·nH_2_O, the value decreased as a function of reaction time, which results from a fast degradation of HMF over this catalyst. It is worth mentioning that niobic acid was also very active in glucose conversion. In fact, the conversion of substrate obtained by Nb_2_O_5_·nH_2_O in the first 30 min of the reaction (63%) was higher than that obtained by Nb_2_O_5__550_air (50%) and Nb_2_O_5__550_N_2_ (47%) in 90 min. 

Moreover, as shown in Figure 10, the concentration of fructose decreased with time, which supports the two-step reaction mechanism involving subsequent isomerization and dehydration reactions, which was also the case when Nb_2_O_5__SA was used (see Figure 9). Interestingly, published data dealing with the application of niobium based catalysts in glucose dehydration in general report very low selectivity to fructose (e.g., <4%), which was attributed to the fast dehydration of fructose on the BA sites of the respective catalysts [15,38,68]. Hara et al. even concluded that the observed small amounts of fructose may not be related to the production of HMF at all [69]. However, under our reaction conditions, all niobium oxide materials showed high selectivity towards fructose (i.e., 18–41% in the first 30 min, and 12–30% in 90 min), with the lowest values obtained by the most acidic material (i.e., Nb_2_O_5_·H_2_O) and highest values measured in the case of crystalline catalysts. These observations suggest that the dehydration of fructose takes place with prior desorption of fructose and its subsequent readsorption on the surface of the catalysts as a result of low amount of available BA sites especially on the surface of the crystalline materials studied here. Due to an insufficient amount of BA sites on these catalysts, the isomerization reaction became predominant in the cascade process, which resulted in the accumulation of fructose in the reaction mixture. Thus, it seems that the crystalline niobium oxide catalysts studied here could be considered mostly as LA catalysts. Nevertheless, the selectivity to HMF obtained over these materials was higher than the selectivity to fructose, both at the beginning and after 90 min of the reaction, which means that the sites facilitating fructose conversion to HMF (i.e., BA sites) were also present on the surface of these materials. Concerning the niobic acid, since it had the highest total acidity among all materials studied here, it also had more BA sites available for fructose dehydration, which resulted in lower accumulation of this intermediate in the reaction mixture. Nevertheless, the increase of HMF on this catalyst was not proportional to the decrease in fructose concentration, suggesting that that large amount of fructose was converted to humins and/or other unidentified side products.

It is surprising to note that even the materials that were treated at high temperatures and as such containing very low total acidity still showed some activity in this cascade process, especially in the second step of the reaction, namely fructose dehydration to HMF, which requires BA sites. It should be taken into account that with the increasing calcination temperature, the proportion of the LA compared to the BA sites is higher [15], which is also connected to the fact that variable surface LA sites can be created at elevated temperatures on the surface of Nb_2_O_5_ catalysts by the transformation of protonic sites to LA sites with the elimination of water [43]. Thus, the treatment of Nb_2_O_5_·nH_2_O at 550 °C (to obtain Nb_2_O_5__550_air and Nb_2_O_5__550_N_2_) caused not only the elimination of some acid sites, but also an increase in the proportion of the LA as compared to the BA sites [43]. However, these catalysts, apart from large selectivities to fructose, also showed moderate yields of HMF. Consequently, the catalytic results depicted in Figure 10 suggest that LA sites could also be active in dehydration of fructose to HMF. In fact, it was previously observed that the dehydration of glucose to HMF was catalyzed by niobic acid containing only LA sites, with BA sites blocked with Na^+^ [33,73]. In another study, dealing with fructose dehydration to HMF using niobium phosphates, it was evidenced that catalysts containing mostly LA sites were able to catalyze the dehydration of fructose to HMF; however, these catalysts were less efficient than their counterparts containing BA sites [66]. Conversely, it should be taken into account that, in the present work, the reaction was carried out in water and, in such an environment, niobium oxide materials are able to restore some of their BA sites by rehydration [23,33,38,39]. Thus, taking into account the very low total acidity of the crystalline materials studied here (see niobium oxide treated at 550 °C and Nb_2_O_5__SA in Table 2), it is more likely that the activity of these catalysts in the cascade reaction is due to the regenerated BA sites, rather than the result of the activity of LA sites in the dehydration reactions. 

Last but not least, comparing the catalytic performance within only the crystalline samples (i.e., Nb_2_O_5__SA, Nb_2_O_5__550_air and Nb_2_O_5__550_N_2_), very similar yields of HMF were obtained with all these materials (see Figure 10) in the first 30 min of the reaction, regardless of their total acidity (see Table 2), specific surface area (S_BET_) or the type of gas used during thermal treatments. Nevertheless, in 90 min of the reaction, Nb_2_O_5__SA showed substantially lower selectivity to HMF accompanied with a higher conversion of glucose as compared to the samples obtained from niobic acid via thermal treatment (i.e., Nb_2_O_5__550_air and Nb_2_O_5__550_N_2_). This difference in catalytic performance can be attributed to a different exposure of the active sites on the surface of these catalysts as a result of varied niobium oxide crystalline phases present (i.e., only TT phase in case of Nb_2_O_5__550_air and Nb_2_O_5__550_N_2_ and a combination of TT and H in case of Nb_2_O_5__SA). On the other hand, comparing the influence of the atmosphere used during thermal treatments of Nb_2_O_5_·nH_2_O, it can be seen in Figure 10 that in the first 30 min of the reaction substantially higher selectivity to HMF was obtained over the catalyst treated in air (i.e., Nb_2_O_5__550_air), which also showed higher acidity than its counterpart treated in nitrogen flow (i.e., Nb_2_O_5__550_N_2_). However, in the extended reaction time, both catalysts showed very similar behavior and only slightly higher yield of HMF was obtained using Nb_2_O_5__550_air.

## 4. Conclusions

Different niobium oxide catalysts obtained by thermal treatments of niobium acid were tested in glucose dehydration to HMF, in order to find the influence of these treatments on their acidity and in turn on their catalytic performance in one-pot cascade dehydration of glucose to HMF in water. Increasing the temperature of the thermal treatment brought about a parallel destruction of the texture and decrease in the total acidity of the niobium oxide materials. The catalytic tests revealed that the best performing catalyst was niobic acid calcined at 300 °C, which achieved a substantial yield of 37% of HMF with 66% conversion of glucose in 90 min of an environmentally friendly one-pot process. The achieved results are highly competitive with currently reported aqueous dehydration systems using glucose as feedstock, especially taking into account the simplicity of the applied approach, which does not require the use of extracting solvents or any other additives/promoters. Moreover, the studied niobium oxide catalysts showed almost no activity in rehydration of HMF to formic and levulinic acids, which helped in sustaining a high selectivity to the target product. In general, amorphous niobic acid (Nb_2_O_5_·nH_2_O), which displayed the highest total acidity, was very active in fructose dehydration to HMF, however, only in the shorter reaction time. Over an extended time, it largely facilitated the condensation/polymerization of HMF to form humins. On the other hand, niobic acid treated at elevated temperatures (i.e., 550 °C), displayed very low total acidity, and was found to be a better catalyst for glucose isomerization to fructose, showing only limited activity in fructose dehydration to HMF mainly due to BA sites created by rehydration in water. Thus, it was found that only thermal treatment of niobic acid at an intermediate temperature of 300 °C allowed to attain an adequate value of acidity on its surface, which led to acceptable glucose conversions accompanied with low degradation of HMF to side products. Thus, it was evidenced that only the removal of a suitable part of acidic sites from the surface of highly acidic niobic acid allows improved stability of HMF to be obtained even in pure water.

## Figures and Tables

**Table 1:** Textural properties of the materials studied.

Sample	S_BET_ (m^2^/g)	S_meso_ (m^2^/g)	V_tot_ (cm^3^/g)
Nb_2_O_5_·nH_2_O	366	366	0.27
Nb_2_O_5__300_air	209	209	0.23
Nb_2_O_5__550_air	27	27	0.20
Nb_2_O_5__550_N_2_	39	39	0.13
Nb_2_O_5__SA	4	4	0.12

**Table 2:** The atomic abundance of Nb and O calculated from X-ray photoelectron spectroscopy (XPS) survey spectra, together with the total acidity of the samples obtained from a titration method.

Sample	Nb3d (At%)	O1s (At%)	O/Nb	A_tot_ (mmol/g)
Nb_2_O_5_·nH_2_O	25.76	74.24	2.88	2.35
Nb_2_O_5__300_air	25.97	74.03	2.85	1.69
Nb_2_O_5__550_air	26.61	73.39	2.76	0.19
Nb_2_O_5__550_N_2_	26.69	73.31	2.75	0.05
Nb_2_O_5__SA	26.81	73.19	2.73	0.02

**Table 3:** Comparison of the contribution of different oxygen species to O1s XPS regions of the studied samples.

Sample	O(I) (%)	O(II) (%)	O(III) (%)	O(II)/O(I)
Nb_2_O_5_·nH_2_O	83.96	10.08	5.96	0.12
Nb_2_O_5__300_air	83.26	11.33	5.41	0.14
Nb_2_O_5__550_air	86.84	8.18	4.98	0.09
Nb_2_O_5__550_N_2_	85.81	8.96	5.23	0.10
Nb_2_O_5__SA	85.26	9.80	4.94	0.11

**Table 4:** Temperatures of the maximum NH_3_ desorption and the relative contribution (%) of acid sites calculated from NH_3_-TPD results.

Sample	T_max_ (°C)	Weak (%)	T_max_ (°C)	Medium (%)	T_max_ (°C)	Strong (%)
Nb_2_O_5__SA		0	325	53	410	47
Nb_2_O_5__300_air	270	22	315	33	394	45

## Supplementary Materials

The following are available online at https://www.mdpi.com/2079-4991/10/9/1685/s1, Figure S1: Correlation of the S_BET_ of the catalysts with the obtained yield of HMF in the initial 30 min of the reaction; Figure S2: Correlations of selectivity to fructose vrs total acidity and vrs S_BET_ (A) and (B) in 30 min reaction (C) and (D) in 90 min of the reaction (Reaction conditions: 30 mL of 1.5 wt% solution of glucose in UP H2O, *p* = 2.5 bar of N2, 0.1 g of a catalyst, T = 180 °C, stirring speed = 400 rpm); Figure S3: Selectivity to fructose vrs O/Nb atomic ratio in (A) 30 min of the reaction and (B) 90 min of the reaction (Reaction conditions: 30 mL of 1.5 wt% solution of glucose in UP H_2_O, *p* = 2.5 bar of N_2_, 0.1 g of a catalyst, T = 180 °C, stirring speed = 400 rpm).
