# Supplementary material for: Impact of Thermal Treatment of Nb2O5 on Its Performance in Glucose Dehydration to 5-Hydroxymethylfurfural in Water
Source: Nanomaterials (Basel). 2020 Aug 27;10(9):1685. doi: 10.3390/nano10091685 (PMC7559716; doi:10.3390/nano10091685)
Supplement: Supplementary file 1 [file nanomaterials-10-01685-s001.pdf]

SUPPORTING MATERIALS

Impact of Thermal Treatment of Nb_2O_5 on Its Performance in Glucose Dehydration to 5-Hydroxymethylfurfural in Water

Katarzyna Morawa Eblagon [1,*], Anna Malaika [2,*], Karolina Ptaszynska [1,2], Manuel Fernando R. Pereira [1] and José Luís Figueiredo [1]

[1] Associate Laboratory LSRE-LCM, Faculty of Engineering, University of Porto, Rua Dr. Roberto Frias s/n, 4200-465 Porto, Portugal; karolina.ptaszynska@amu.edu.pl (K.P.); fpereira@fe.up.pt (M.F.R.P); jlfig@fe.up.pt (J.L.F.)

[2] Faculty of Chemistry, Adam Mickiewicz University in Poznań, Uniwersytetu Poznańskiego 8, 61-614 Poznań, Poland

* Correspondence: keblagon@fe.up.pt (K.M.E.), amalaika@amu.edu.pl (A.M.)

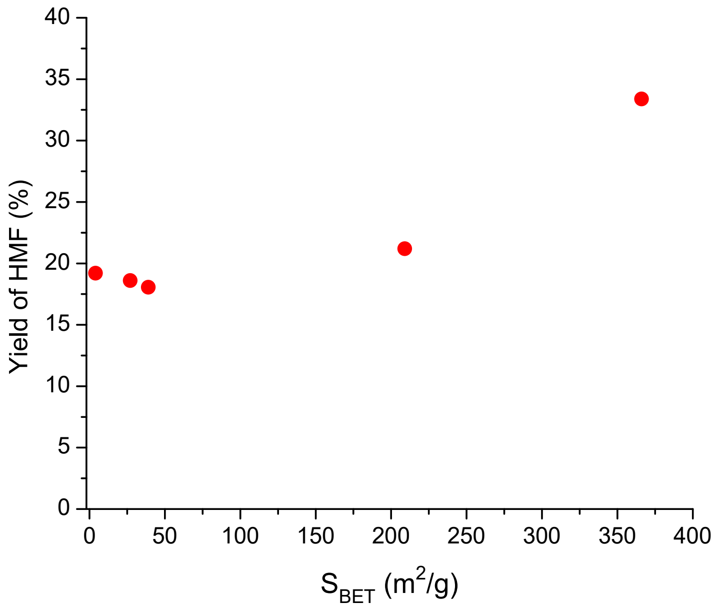

Figure 1. *Correlation of the S_{BET} of the catalysts with the obtained yield of HMF in the initial 30 min of the reaction.*

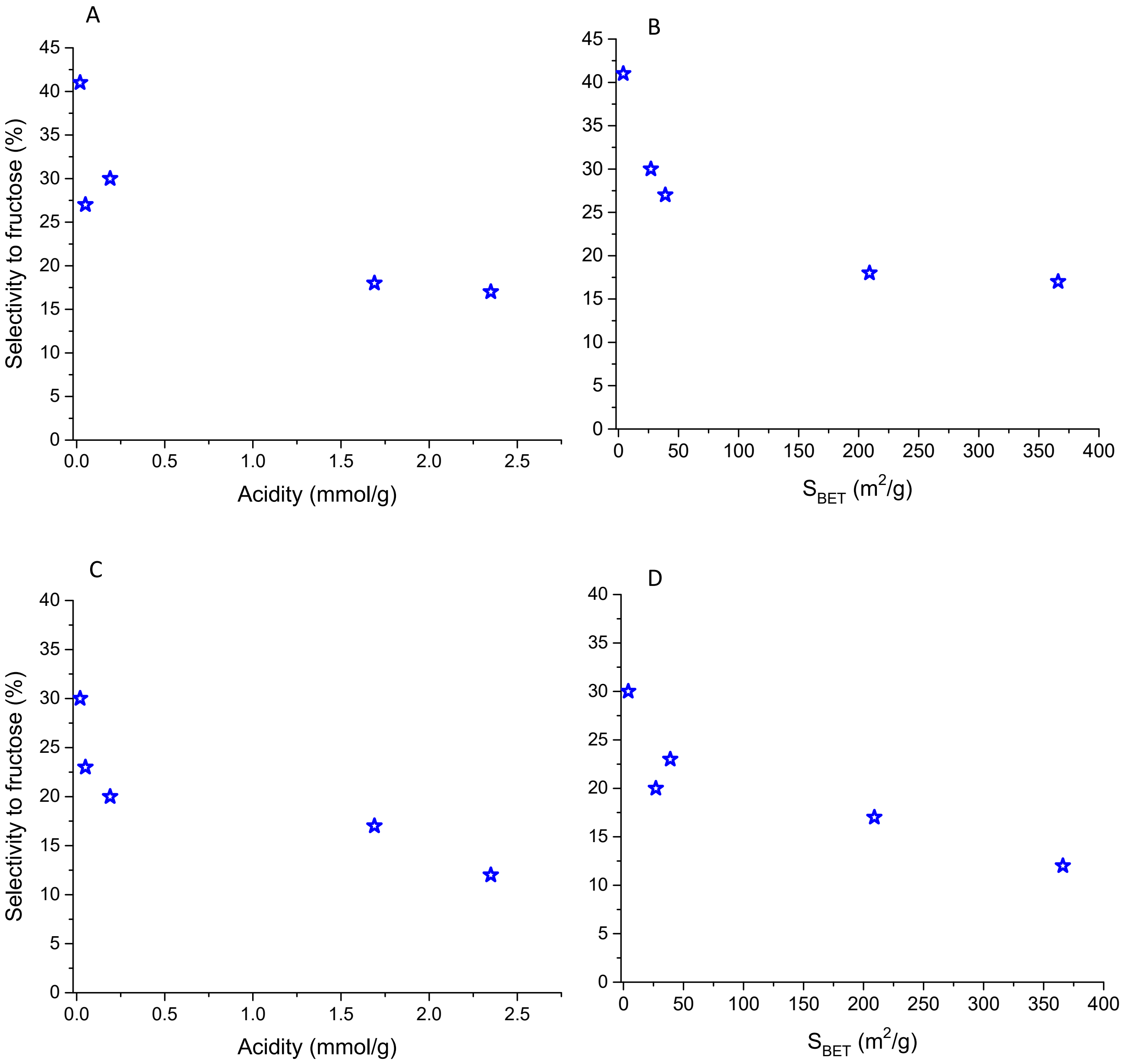

Figure S1.*Correlations of selectivity to fructose vrs total acidity and vrs S_{BET} A) and B) in 30 min reaction C) and D) in 90 min of the reaction (Reaction conditions: 30 mL of 1.5 wt% solution of glucose in UP H2O, p = 2.5 bar of N2, 0.1 g of a catalyst, T = 180 °C, stirring speed = 400 rpm).*

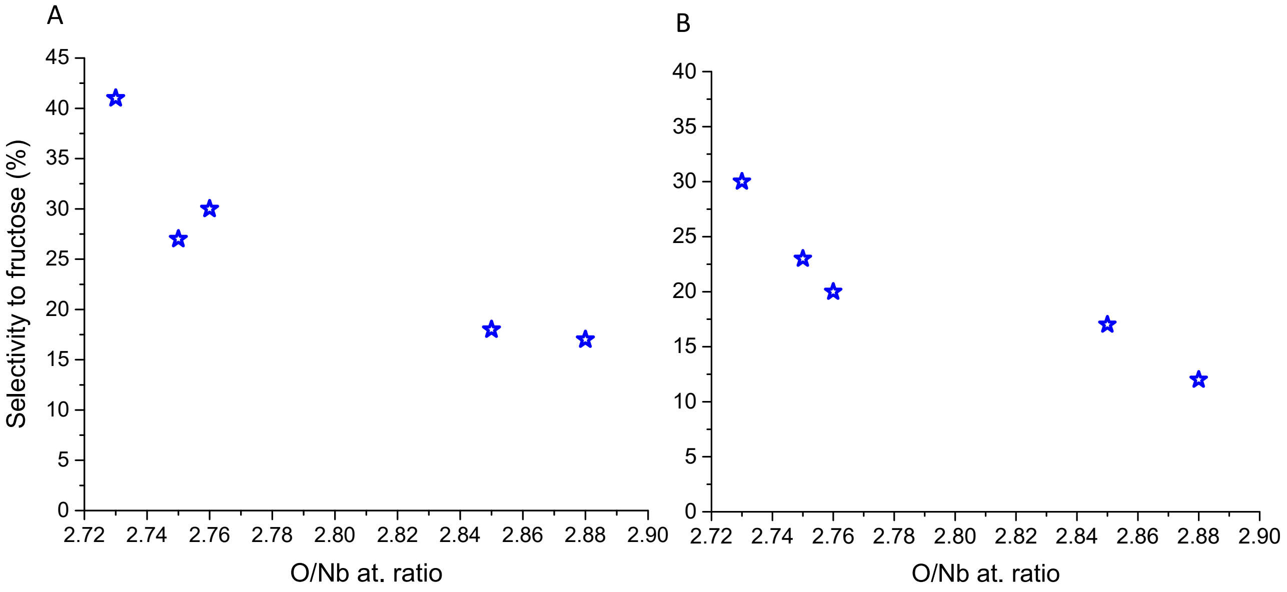

Figure S3. *Selectivity to fructose vrs O/Nb atomic ratio in A) 30 min of the reaction and B) 90 min of the reaction (Reaction conditions: 30 mL of 1.5 wt% solution of glucose in UP* H_2O*, p = 2.5 bar of* N_2*, 0.1 g of a catalyst, T = 180 °C, stirring speed = 400 rpm).*
